# Health Status Assessment of Panamanian Pacific Coastal Areas Using Multi-Level Biomarkers in the Mangrove Cockle *Anadara tuberculosa*

**DOI:** 10.3390/toxics14080710

**Published:** 2026-08-12

**Authors:** Nelva Elena Alvarado-González, Jenifer Ortega, Yulissa De Gracia, Maricselis Díaz, Enrique Medianero, Tifanie Briaudeau, Tamer Hafez, Maren Ortiz-Zarragoitia, Xabier Lekube, Beñat Zaldibar

**Affiliations:** 1 Instituto Especializado de Análisis (IEA), Vicerrectoría de Investigación y Postgrado, Universidad de Panamá, Panama City 0824, Panama; nelva.alvarado@up.ac.pa; 2 Centro Regional Universitario de Azuero, Universidad de Panamá, Panama City 0824, Panama; ajuliette2913@gmail.com (J.O.); yulissa041221@gmail.com (Y.D.G.); 3 Autoridad de los Recursos Acuáticos de Panamá (ARAP), Panama City 0824, Panama; 4 Maestría en Gestión Ambiental y Sostenibilidad, Universidad del Istmo, Panama City 0796, Panama; 5 Centro de Investigaciones Psicofarmacológicas, Facultad de Medicina, Universidad de Panamá, Panama City 0824, Panama; maricselis.diaz@up.ac.pa; 6 Miembro del Sistema Nacional de Investigación (SNI-SENACYT), Panama City 0816, Panama; 7 Departamento de Ciencias Ambientales, Facultad de Ciencias Naturales, Exactas y Tecnología, Universidad de Panamá, Panama 3366, Panama; 8 CBET + CRG, Department of Zoology and Animal Cell Biology, Faculty of Science and Technology, University of the Basque Country (UPV/EHU), 48940 Leioa, Biscay, Spain; tifanie.briaudeau@ehu.eus (T.B.); tamer.hafez@ehu.eus (T.H.); maren.ortiz@ehu.eus (M.O.-Z.); xabier.lekube@ehu.eus (X.L.); 9 Research Centre for Experimental Marine Biology and Biotechnology (PIE), University of the Basque Country (UPV/EHU), 48620 Plentzia, Biscay, Spain

**Keywords:** Panama, monitoring, pollution, mollusc, biomarkers, histopathology

## Abstract

Tropical coastal ecosystems are highly productive but increasingly threatened by anthropogenic pressures, making environmental monitoring tools essential to determine the health status of the environment. In this study, the health status of three sites along the Pacific coast of Panama (Río Santa María, Cuchilluyo, and Montijo) was assessed using the bivalve mollusc *Anadara tuberculosa* as a sentinel species. A multilevel biomarker approach was employed, combining chemical analyses with biochemical and histological biomarkers. Metal concentrations in whole soft tissues were generally low-to-moderate, except for arsenic, and polycyclic aromatic hydrocarbon (PAH) concentrations were low in all sites, being dominated by low-molecular-weight compounds. Biomarker responses were consistent with the chemical data, with cockles from Montijo exhibiting lower glutathione S-transferase (GST) activity and reduced oxidative DNA damage. Histological analyses revealed only mild alterations, including hemocytic infiltration in the connective tissue of digestive glands and atrophy, with no major reproductive impairments. Overall, results indicate low to moderate pollution levels in the studied areas, with Montijo representing the least impacted site. The study supports the suitability of *A. tuberculosa* as a sentinel species and the relevance of a multilevel biomarker approach for assessing environmental health in tropical coastal ecosystems.

## 1. Introduction

Coastal ecosystems of tropical regions are among the most productive and biodiverse environments [1], yet they are highly vulnerable to diverse anthropogenic pressures [2]. The Republic of Panama is located between the Caribbean Sea and the Pacific Ocean and hosts a high biodiversity of aquatic ecosystems, including both freshwater and marine water environments [3,4]. However, its Pacific coast is subject to significant pollutant inputs, particularly during the rainy season when riverine systems transport land-based runoff into coastal waters. In addition, the strategic importance of the Panama Canal results in intense maritime traffic, introducing additional risks of hydrocarbon and heavy metal contamination [5]. Although limited information is available in the specific case of the Pacific coast of Panama, previous studies have indicated the presence of pollutants such as heavy metals in the sediments [6,7] and biota [8] as well as a significant amount of plastic debris [9].

In this study, two coastal areas characterized by different levels of anthropogenic pressures were investigated. The Parita Bay (7°57′18″–8°18′40″ N 80°13′37″–80°21′49″ W), located on the central Pacific coast of Panama, is part of the country’s “Dry Arc” (the driest zone) region. The study area includes five watersheds and is characterized by dry tropical and premontane forests. The bay includes the Sarigua National Park and the Cenegón de Mangle Wildlife Refuge, which were established in 1984 and 1980, respectively. Notably, more than 50% of the protected area is used as shrimp-breeding ponds [10]. The Parita Bay limits with Coclé and Herrera provinces and agriculture, livestock, hunting, forestry, transportation, storage, and communications are the most important sectors. In addition, mining operations, energy infrastructures, tourism facilities, and grain processing plants are identified as productive facilities [11]. The main urban area of the region is Chitré, with 100,000 people. Recently it has been described as a growing concern regarding the La Villa River due to increasing pollution problems in relation to agricultural and livestock activities [12]. On the other hand, the Gulf of Montijo (7°56′4″–7°59′30″ N & 81°17′33″–81°1′40″ W) is a protected wetland area of international importance (Site Ramsar). It presents a rich aquatic environment of estuaries, deltas, beaches, muddy and sandy areas, and extensive mangrove forests. It is an important fishing area in the Panamanian Pacific with multispecies fisheries, including bivalves, crustaceans, fish, and polychaetes [13]. Accordingly, main economic activities are related to fishermen and shellfish extractors, but also agriculture is relevant in the area [6]. In the Montijo Gulf previous studies have indicated the presence of Cd in sediments [7], but in previous studies bivalves collected in Montijo have shown low levels of pollutants [8].

Bivalve molluscs have been used as sentinel organisms of the environment for several decades due to their filter-feeding behavior, sessile lifestyle, and high bioaccumulation capacity [14,15]. Concentration of pollutants in bivalve tissues reflects both the presence and availability of these compounds in the environment. *Anadara tuberculosa* is a filter-feeding bivalve mollusc endemic to Pacific mangroves of the Americas. It develops mainly in muddy substrates, especially clays and clayey silts [16], and has been proposed as a relevant sentinel bivalve species in Nicaragua, Ecuador, or Costa Rica [17,18,19]. In addition, *A. tuberculosa* is widely consumed by local populations along the Central American Pacific coast, making it a species of considerable socioeconomic importance [13,20,21] but also a potential pathway for human exposure to environmental contaminants due to its bioaccumulation ability. Among these contaminants, toxic metals and polycyclic aromatic hydrocarbons (PAHs) are of particular concern due to their persistence, bioaccumulation in edible tissues, and potential carcinogenic and non-carcinogenic effects [22,23]. Consequently, estimating dietary intake and associated health-risk indices—such as target hazard quotient (*THQ*), hazard index (*HI*), and cancer risk—provides an important link between environmental contamination and seafood safety [22,24]. This approach is especially relevant in regions where contaminant-monitoring programs and local consumption data are scarce, such as parts of the Pacific coast of Panama, and can contribute to identifying vulnerable areas, supporting risk management, and improving the protection of coastal populations dependent on mangrove bivalves.

Environmental risk assessment studies include biomarker measurements as sensitive indicators of sublethal effects caused by environmental stressors in the marine biota, including molluscs [25,26,27,28]. Biomarkers can be molecular, subcellular, cellular, and physiological alterations. In the present study, chemical measurements and biomarkers of different biological organization levels (biochemical, cellular, and tissue-level) were assessed to determine the general health status of 3 sites along the Pacific coast of Panama: Rio Santa María, Cuchilluyo, and Montijo. The bioaccumulation of metals and PAHs contaminants was determined through chemical analysis of whole-body samples. Molecular alterations were assessed by measuring DNA oxidative damage after 8-Oxo-7,8-dihydro-2′-deoxyguanosine (8-oxodG) immunohistochemistry (IHC). At the sub-cellular level, catalase (CAT), glutathione S-transferase (GST), and acetylcholinesterase (AChE) activities were used as indicators of oxidative stress, detoxification processes, and neuronal activities, respectively. Finally, histopathological analyses were carried out to assess tissue-level alterations.

## 2. Materials and Methods

### 2.1. Sample Area and Strategy

**Figure 1:**
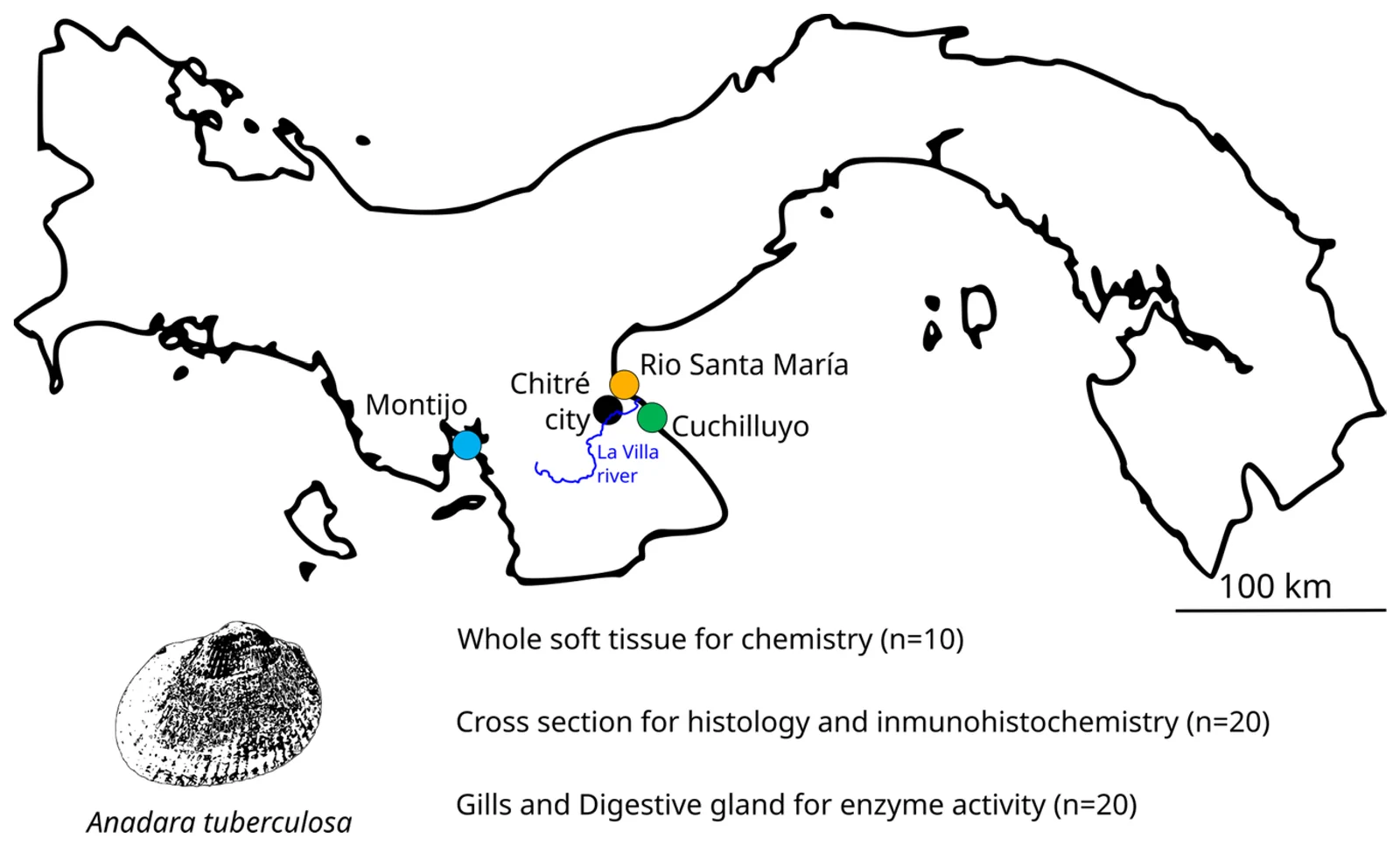
Map of Panama showing sampling sites and tissue samples collected.

Two primary study areas were established: (1) the eastern Azuero Peninsula within the Parita Bay encompassing two sampling sites (Rio Santa Maria and Cuchilluyo); and (2) the western part of the Azuero Peninsula within the Gulf of Montijo (Figure 1). Bivalve samples (*n* = 30 per site) were collected by local fishermen in the first week of May 2024 in all sites. Specimens collected in Rio Santa María and Cuchilluyo were transported to Puerto Parita for immediate dissection, while those collected in Montijo were transported in ice to the laboratory (Panama City) and immediately dissected.

### 2.2. Chemical Measurements

For chemical measurements, the soft tissues of 10 bivalves of each site were dissected out, pooled, homogenized, and freeze-dried before being stored at −20 °C. Metals (Ag, Al, As, Cd, Co, Cr, Cu, Fe, Hg, Mn, Ni, Pb, Se, V, and Zn) were measured according to [29,30] by Inductively Coupled Plasma-Mass Spectrometry (ICP-MS; NexION 300 Perkin Elmer, Shelton, CT, USA) after microwave digestion (Multi-wave 3000, Anton Paar, Graz, Austria). Polycyclic Aromatic Hydrocarbons (PAHs) were analyzed by Gas Chromatography-Mass Spectrometry (GC–MS), as detailed by [15]. The chemical measurements were performed by the SGIKER Service at the University of the Basque Country (EHU).

The estimated daily intake of heavy metals and PAHs for human consumption was calculated for adult males according to the following Formula (1):

(1) $$EDI=\frac{C\times FIR}{BW}$$

where *EDI* is the estimated daily intake for adults through a bivalve diet (µg/kg/day); C represents the concentration of the compounds (µg/g wet weight); FIR is the daily intake of bivalves based on the consumption of *A. tuberculosa* in Ecuador (no data is available for Panamá) established at 157.14 g/day [31]; and BW is the average body weight for an adult established at 70 kg.

The target hazard quotient (*THQ*), established as a combined risk index for non-carcinogenic compounds, was calculated as follows (2):

(2) $$THQ=\frac{C\times FIR\times EF\times ED}{BW\times RfD\times AT}\;\times \;10^{\;-\;3}$$

where EF is the exposure frequency (350 days/year); ED is the exposure duration (70 days); AT is the mean exposure duration for non-carcinogens [32]; and RfD is the oral reference dose with values of 0.04 (Cu); 0.001 (Cd); 0.003 (Cr); 0.3 (Zn); 0.0001 (Hg); 0.00015 (Pb); 0.02 (AcP); 0.0003 (BaP); 0.04 (Flu); 0.04 (FL); 0.3 (Ant); 0.03 (Pyr) and 0.02 (NaP) mg/kg/day [22,33].

The total sum of calculated *THQ*s was used to obtain the total hazard index *HI*, according to the following Formula (3):

(3) $$HI=\sum_{i=1}^{n}THQ\;i$$

Finally, the cancer risk (*TR*) was calculated according to the following Equation (4):

(4) $$TR=\frac{C\times FIR\times EF\times ED}{BW\times RfD\times AT}CSF\;\times \;10^{\;-\;3}$$

where *CSF* is the carcinogenic slope factor: The *CSF* values were 1.5 (As); 6.3 (Cd); 0.5 (Cr); 0.0085 (Pb); 7.3 (BaP and DBA); 0.73 (BaA, BbFL, and Ind); 0.073 (BkFL) and 0.0073 (Chr) [20,22]. According to [22]; *TR* values > 1 × 10^−4^ indicates carcinogenic risk; values 1 × 10^−6^ < *TR* < 1 × 10^−4^ indicate acceptable carcinogenic risk, and values *TR* < 1 × 10^−6^ indicate negligible carcinogenic risk. In the case of As, the risk corresponds to the inorganic arsenic, and for the present calculations, it has been considered that inorganic arsenic constitutes 3% of the total arsenic [34,35].

### 2.3. Biochemical Measurements

Digestive glands and gills of another 20 bivalves per site were dissected out (*n* = 20) and homogenized (0.1 M potassium phosphate buffer, pH 7.4, at ratios of 1:4 for digestive gland and 1:5 for gills) and centrifuged (30 min, 12,000× *g*, 4 °C) prior to enzymatic activity measurements. The post-mitochondrial supernatant (PMS) was used to determine catalase (CAT), glutathione-S-transferase (GST), and acetylcholinesterase (AChE) activities using a BioTek Eon microplate spectrophotometer (BioTek Instruments, Highland Park, IL, USA). Enzyme activities were expressed as a function of the protein concentration in the samples, which was determined according to Bradford’s method adapted to microplate and using bovine serum albumin as a standard [36].

AChE activity was determined at 412 nm, using Ellman’s colorimetric method adapted to microplate [30], and expressed as specific activity in nmol DTNB/min/mg prot. GST activity was determined at 340 nm for 6 min, following Habig’s method adapted to microplate [35] and expressed as nmol/min/mg prot. CAT activity was expressed as the absorbance decrease measured at 240 nm, according to the method of [37]. Results were expressed as μmol H_2_O_2_/min/mg prot.

### 2.4. Histological Analysis

From the same animals selected for biochemical measurements, a cross section including digestive gland, gonads, and gills was performed to obtain samples for histological analysis (*n* = 20 per site). Cross sections were fixed in seawater-buffered formaldehyde (4%) for 24 h. Formalin-fixed samples were dehydrated in a graded series of ethanol, cleared, and routinely embedded in paraffin (Leica ASP 300S, Wetzlar, Germany). Paraffin blocks were cut in a rotary microtome (Leica RM 2125RTS, Wetzlar, Germany). 5 µm thick sections were stained with Haematoxylin-Eosin [38] for histopathological examination, and 3 µm thick sections were obtained and stained by means of immunohistochemistry (see below).

Haematoxylin-Eosin-stained histological sections were analyzed using a bright-field light microscope for sex, gonad developmental stages, inflammatory lesions, digestive gland epithelial thinning, and disease conditions. Gonad stage determination was calculated according to [17]. Inflammatory responses and digestive gland epithelial thinning were measured using indices described by [39].

### 2.5. 8-Oxo-7,8-dihydro-2′-deoxyguanosine Immunohistochemistry

8-Oxo-7,8-dihydro-2′-deoxyguanosine (8-oxodG) immunohistochemistry (IHC) was performed according to [40]. 3 μm thick sections obtained in the microtome were collected on pre-silanized slides, dewaxed in xylene, hydrated in a decreasing graded ethanol series, and washed in Phosphate-Buffered Saline (PBS). Sections were immersed in RNAse and Proteinase K, and DNA was denatured with HCl. After rinsing in blocking serum following manufacturer explanations (Vectastain Elite ABC Kit, Vector Laboratories, Newark, CA, USA), sections were incubated with an 8-oxodG-specific primary antibody (Trevigen, Gaithersburg, MD, USA) overnight, and the reaction product was visualized using the Peroxidase Vectastain Elite ABC Kit. Finally, sections were dehydrated in graded ethanol series and mounted in DPX. Samples incubated in PBS without 8-oxodG primary antibody were used as IHC negative controls. Further, image analysis (Image J, National Institute of Health, Bethesda, MD, USA) was applied to quantify 8-oxodG IHC in five fields per digestive gland (20× objective lens). Positive nuclei of the digestive epithelium were selected, and the mean average grey value (*MAG*) for each selected area and the area surface (*AS*) were recorded to calculate 8-oxodG optical density (*OD*; arbitrary units) as follows (5):

(5) OD=MAG × AS

### 2.6. Statistical Analyses

Bayesian one-way analyses of variance were conducted to examine the effect of the site. Evidence strength was interpreted using conventional Bayes factor guidelines following [41], where BF_10_ values above 3, 10, and 30 indicate moderate, strong, and very strong evidence, respectively. A Principal Component Analysis (PCA) was completed to highlight differences and contribution weights among sites and studied parameters. All analyses were carried out using the JAMOVI 2.3 Statistics Package [42].

## 3. Results

### 3.1. Biometric Parameters

Cockles from Montijo were larger, with higher length and width, than those collected in Rio Santa María and Cuchilluyo (Table 1). The Bayesian ANOVA also supports the idea of the site-related differences (BF_10_ = 4.0 × 10^6^ and BF_10_ = 8.0 × 10^6^ for length and with, respectively; Table S1). Regarding the length/width ratio, no differences were observed among sites.

### 3.2. Chemical Measurements

Fifteen metals and the priority 16 PAHs of the EPA were measured in the cockles (Table 2). Concentrations of Acenaphthylene, Benzo(b)fluoranthene, Benzo(k)fluoranthene, Benzo(a)pyrene, Benzo(ghi)perylene, and Dibenzoanthracene were below the detection limits in all sites and have not been included in Table 2. Overall, higher metal concentrations were recorded in Rio Santa María and Cuchilluyo compared to Montijo. More detailed Bayesian ANOVA evidence for site-related differences (BF_10_ = 4.57, BF_10_ = 16.95, BF_10_ = 121.19, BF_10_ = 10,080.66, BF_10_ = 5.28, and BF_10_ = 7.96; Table S1) for As, Cd, Hg, Mn, Se, and Zn, respectively. In the case of Cd, Hg, Se, and Zn, the site effect was also strong when Rio Santa María and Montijo are compared (Table S2). Similarly for Hg and As, Cuchilluyo and Montijo are more different sites when pairwise comparisons are performed (Table S2). On the other hand, slightly higher Cr concentrations were observed in Montijo compared to Rio Santa María and Cuchilluyo.

**Figure 2:**
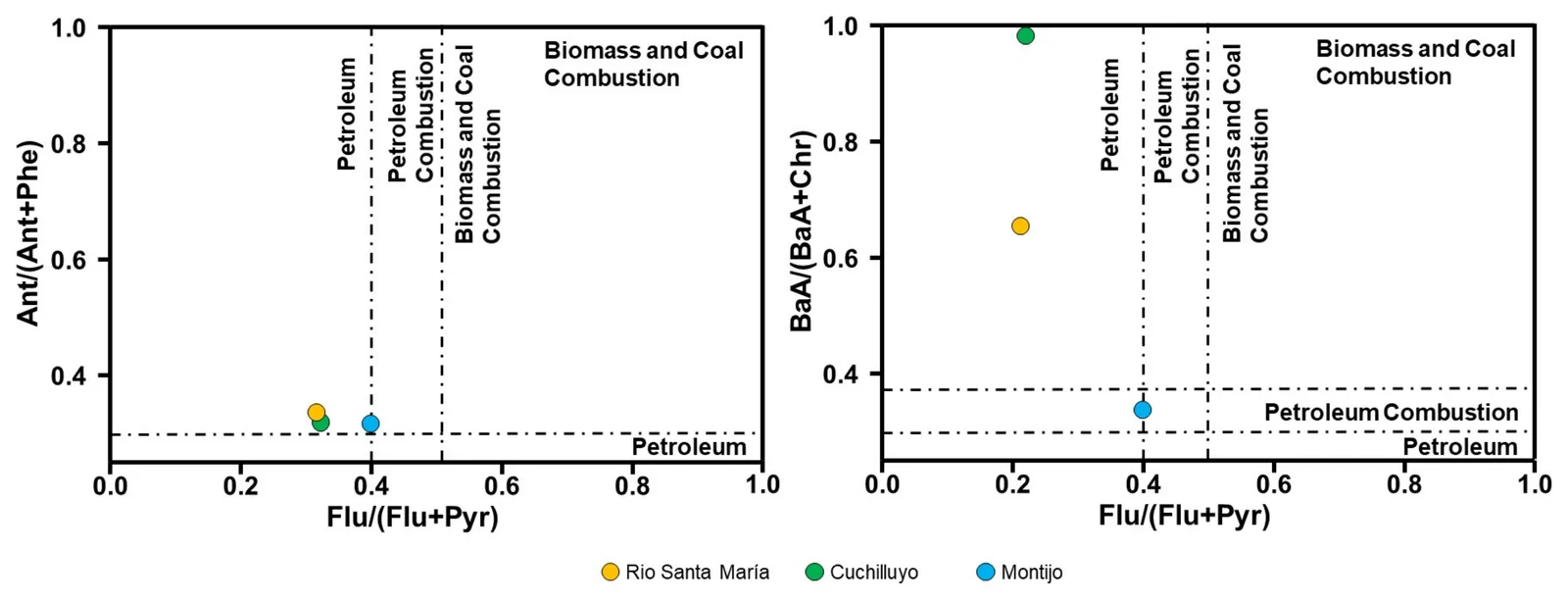
Bivariate cross plots of molecular diagnostic ratios Anthracene/(Anthracene + Phenanthrene) and Fluoranthene/(Fluoranthene + Pyrene) and Benzo(a)anthracene/(Benzo(a)anthracene + Chrysene) in *Anadara tuberculosa* collected in Rio Santa María, Cuchilluyo and Montijo.

In the case of PAHs, almost no relevant differences were recorded among the three sites, and only in the case of Benzo(a)anthracene were higher concentrations measured in Cuchilluyo and, to a lesser extent, in Rio Santa María compared to Montijo (BF_10_ = 4.04), indicating differences among sites (Table S1). The total sum of PAHs indicated some higher levels in Cuchilluyo but without significant differences. LMW/HMW ratios were >1 for all sites with the highest recorded for Montijo (12.22 ± 8.937). This could be mostly related to the relatively high concentration of Fluorene (3 rings) and low concentration of Benzo(a)anthracene (4 rings) (Table 2). In agreement with the LMW/HWM ratio regarding the origin of the PAHs, the combination of the parameters Ant/(Ant + Phe) and Flu/(Flu + Pyr) indicated that PAHs were of petrogenic origin for the three sampling sites. In the case of Montijo, the origin is not that clear since compared to the other two sites, a lower Ant/(Ant + Phe) index and a higher Flu/(Flu + Pyr) are observed. On the other hand, the BaA/(BaA + Chr) ratio is close to or higher than 0.35, indicating that combustion is the dominant source (Figure 2).

*EDI* values for heavy metals were higher for essential elements such as Fe, Zn, or Mn (Table 3). In agreement with heavy metal concentrations recorded, higher levels of *EDI* were measured for Al, As, and Cd in Rio Santa María and Cuchilluyo compared to Montijo. The same trend was observed for *THQ*s of heavy metals, with cockles of Montijo presenting the lowest values and those collected in Rio Santa María and Cuchilluyo presenting similar and higher values. In all cases except for As in Rio Santa María and Cuchilluyo and Zn in all sites, the individual *THQ* values remained below 1. The combined *HI* value was lower in Montijo and higher in Rio Santa María and Cuchilluyo. The *TR* values for carcinogenic elements such as As, Cd, Cr, and Pb indicated negligible carcinogenic risk for Pb in all sites but carcinogenic risk for As, Cd and Cr in all sites (Table 3).

As observed in the concentration data, *EDI* values for organic pollutants were higher for 2 and 3 ring PAHs compared to the heaviest ones (Table 4). Individual *THQ* values all remained below 1 in all sites, and the combined *HI* value was also lower than 1. Regarding carcinogenic substances, only Chr was detected at measurable concentrations with a *TR* value indicating a negligible carcinogenic risk in all sites (Table 4).

### 3.3. Biochemical Measurements

**Figure 3:**
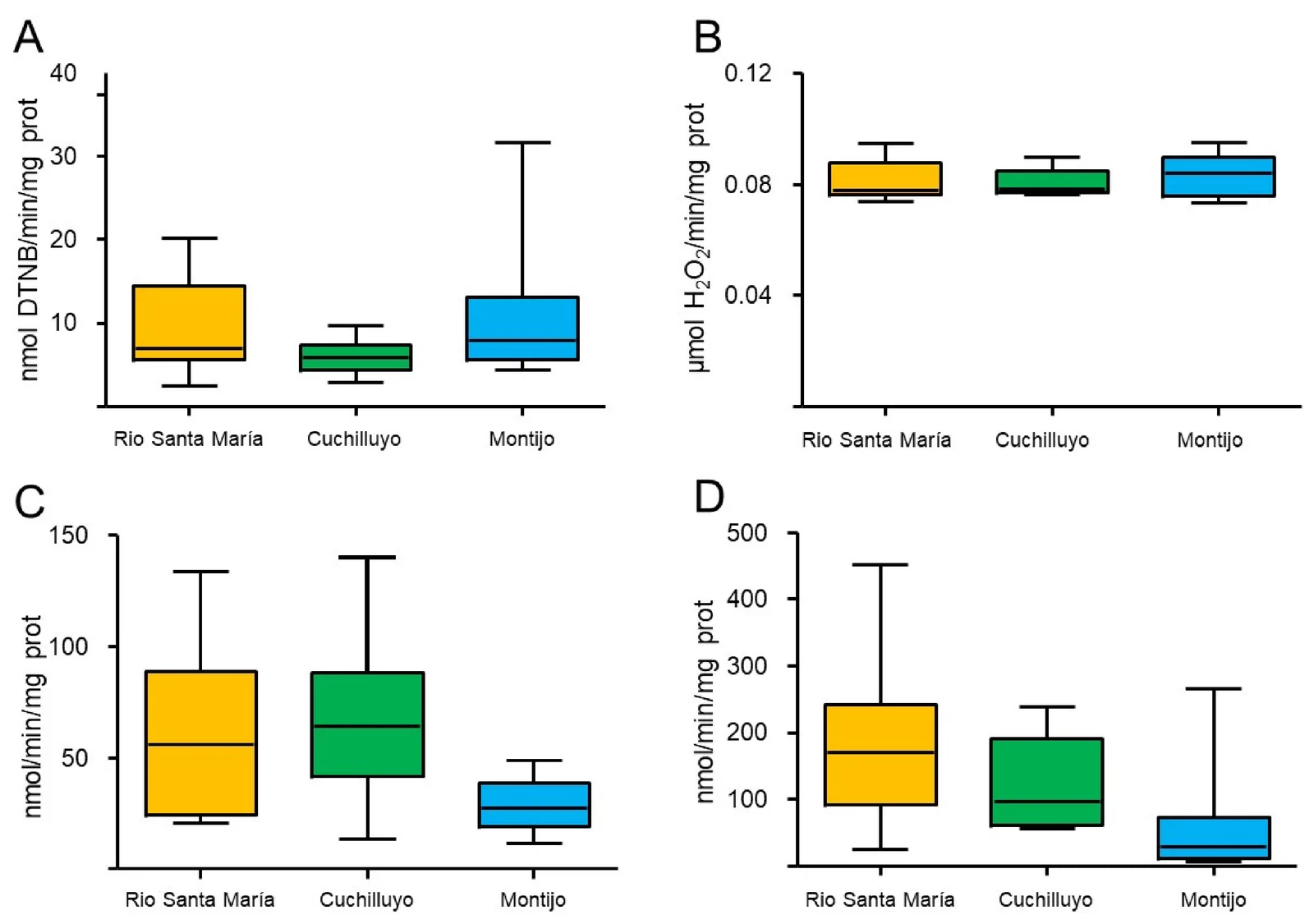
Acetylcholinesterase (**A**), Catalase (**B**), and Glutathione S-Transferase (**C**) activities in gills and Glutathione S-Transferase activity in the digestive gland GST (**D**) of cockles collected in the different sampling sites. Lines indicate the median values, boxes the interquartile values, and whiskers maximum and minimum values.

Regarding biochemical measurements, AChE and CAT activities showed similar levels in all sites (Figure 3) and Bayesian ANOVA evidence for no site-related differences (BF_10_ = 0.39 and BF_10_ = 0.054; Table S1). However, for GST activity, both in gills and digestive glands, more clear trends are observed. In the case of the gills, cockles from Rio Santa María and Cuchilluyo showed similarly higher levels of GST activity (Figure 3C), while in the case of the digestive gland, a more defined trend is observed, with Rio Santa María showing the highest levels, Montijo the lowest, and Cuchilluyo intermediate values (Figure 3D). After Bayesian ANOVA, strong evidence of site effects is observed (Table S1), generally reflecting lower GST activity at Montijo (gill GST: BF_10_ = 21.74; digestive gland GST: BF_10_ = 21.83). Pairwise comparisons supported these differences, particularly between Montijo and eastern and more sites and other sites (Table S2) (Figure 3).

### 3.4. Histological Analysis

**Figure 4:**
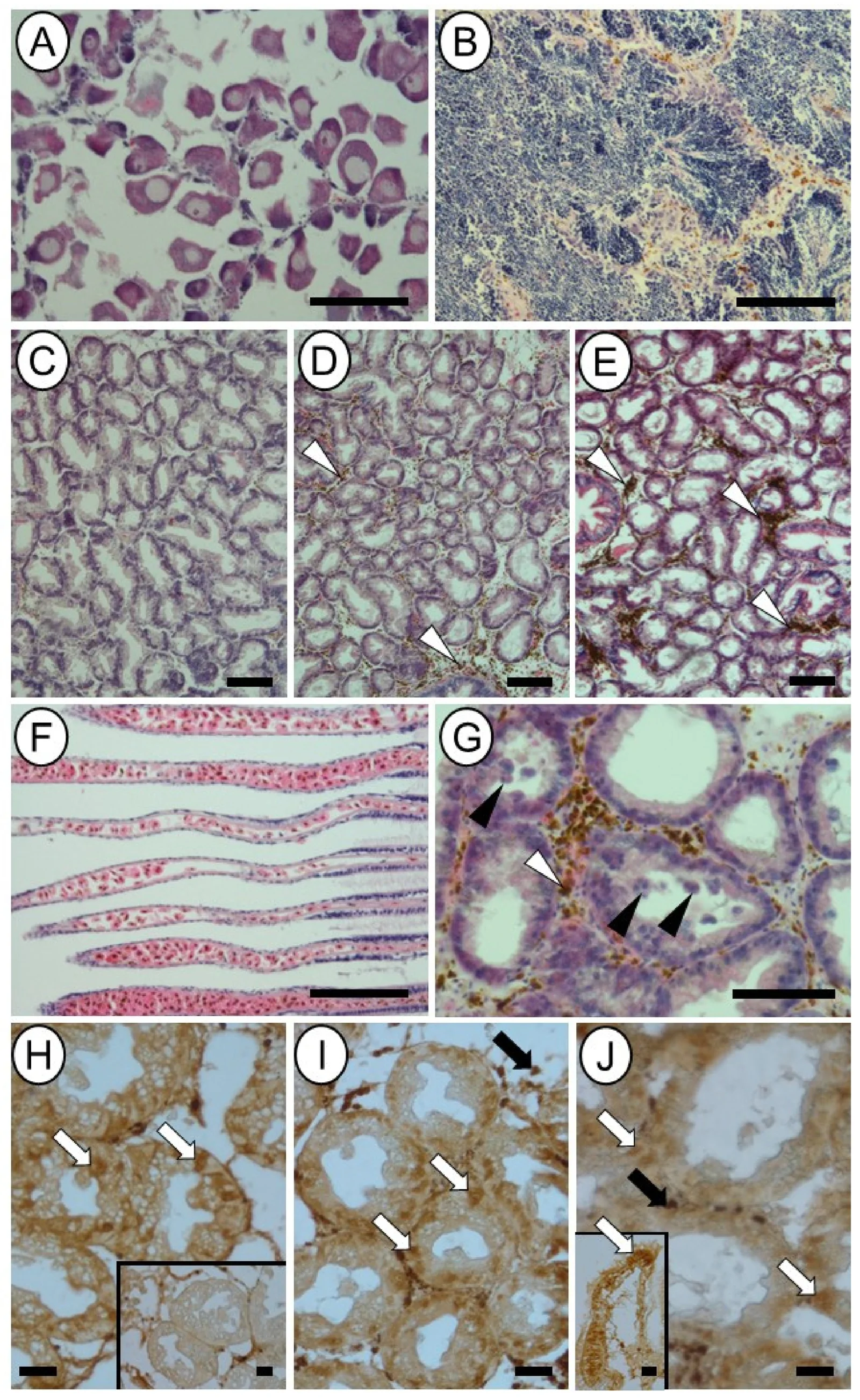
Micrographs of gonad (**A**,**B**), digestive gland (**C**–**E**,**G**–**J**), and gills (**F**) of *Anadara tuberculosa* collected in Rio Santa María (**A**,**C**,**H**), Cuchilluyo (**B**,**D**,**F**,**G**,**I**), and Montijo (**E**,**J**). Note the intense hemocytic infiltrations in the connective tissue of samples from Cuchilluyo and Montijo (white triangles) and the presence of parasites (black triangles) in the digestive tract. The 8-oxodG staining shows the dark brown stained nuclei (white arrows) and the unspecific stain in the hemocytes (black arrows). Note the absence of nuclear stain in the negative control sample (**H** inset). Scale bar = 100 µm (**A**–**G**) and 20 µm (**H**–**J** and inset).

**Figure 5:**
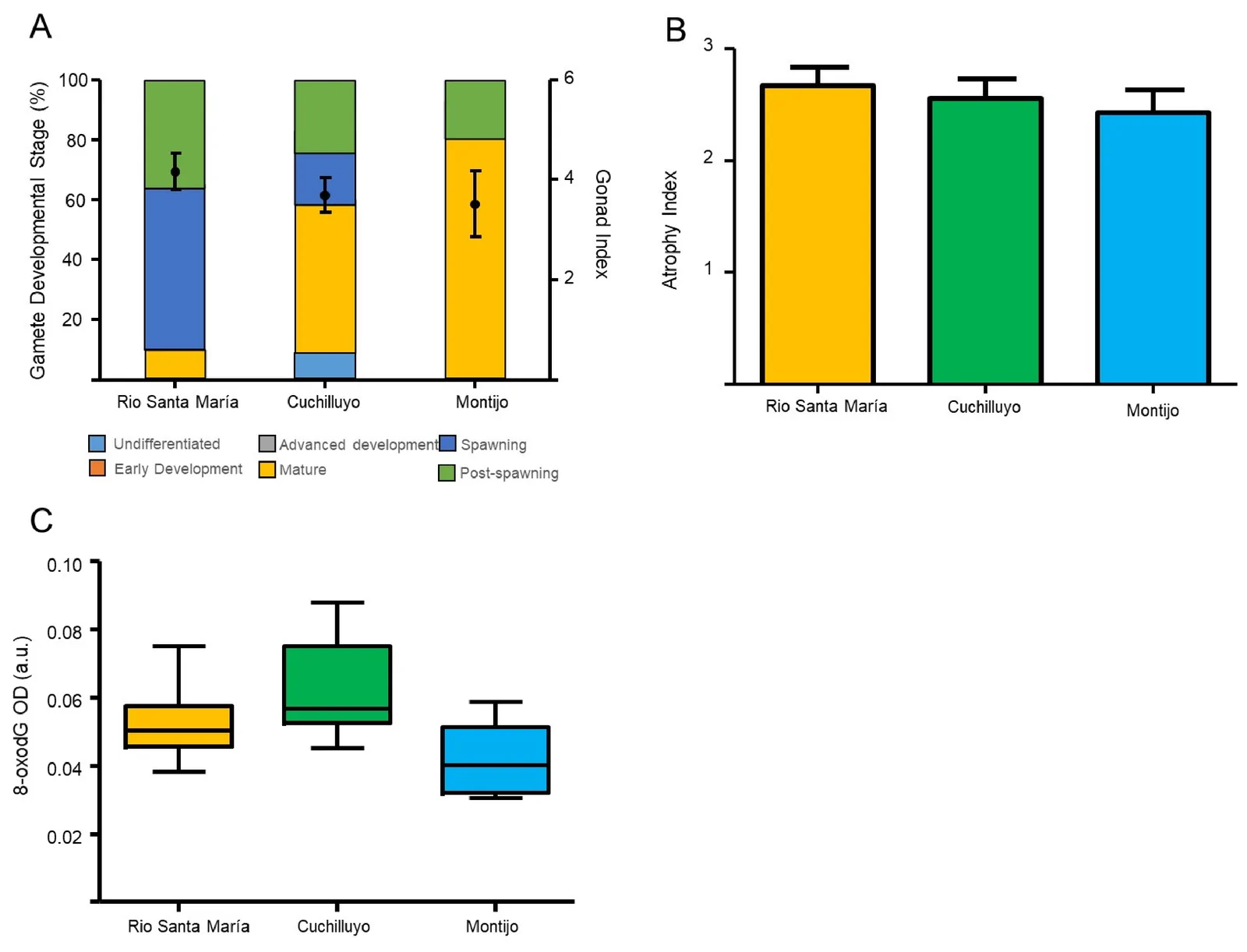
Gamete developmental stages and gonad index (**A**); Atrophy levels (**B**); and 8-oxodG immunohistochemistry staining quantification (**C**) in cockles collected in Rio Santa Maria, Cuchilluyo, and Montijo. In the bar plot means and standard deviations are shown. In the box & whisker plot lines indicate the median values, boxes the interquartile values, and whiskers maximum and minimum values.

Regarding gonad histological analysis, cockles from the three sites presented very similar gamete developmental stages, with most animals in mature or spawning phases (Figure 4A,B and Figure 5A). Overall, no side effects were detected in the gonad index (BF_10_ = 0.089; Table S1), indicating very similar developmental stages. However, some more individuals in spawning and post-spawning phases were observed in Río Santa María.

Few histopathological alterations were observed in studied tissues, with cases of hemocytic infiltrations in the connective tissue of digestive glands and in gill filaments observed in cockles from Cuchilluyo and Montijo (Figure 4F,G). The atrophy index was similar in all sites, with values close to 3, indicating some mild alterations of the digestive gland in all sampling sites. Statistical analysis (Table S1) also showed no site effects on the atrophy index.

Immunohistochemical staining of 8-oxodG was detected in the nucleus of digestive epithelium cells (Figure 4H–J) and gill cells (Figure 4J inset). In the digestive epithelium, digestive cells presented more intense staining, while in the gills, ciliated cells from the frontal area presented higher staining intensity. A strong positive reaction was detected in hemocytes, but since the negative control samples also presented a positive reaction regarding the DNA damage levels, the response of this cell type is not clear and was discarded for the measurements (see Section 4). Therefore, the quantification of the reaction intensity was limited to the digestive gland. Results indicate that cockles from Montijo presented the lowest levels of oxidized guanine (Figure 5C), while higher levels were detected in cockles from Cuchilluyo and intermediate levels were measured in cockles from Rio Santa Maria. ANOVA analysis showed a strong site effect (BF_10_ = 12.65; Table S1), indicating different DNA oxidation levels in the three sites. More specifically, especially different were 8-oxodG levels when Montijo and Cuchilluyo were considered (Table S2).

**Figure 6:**
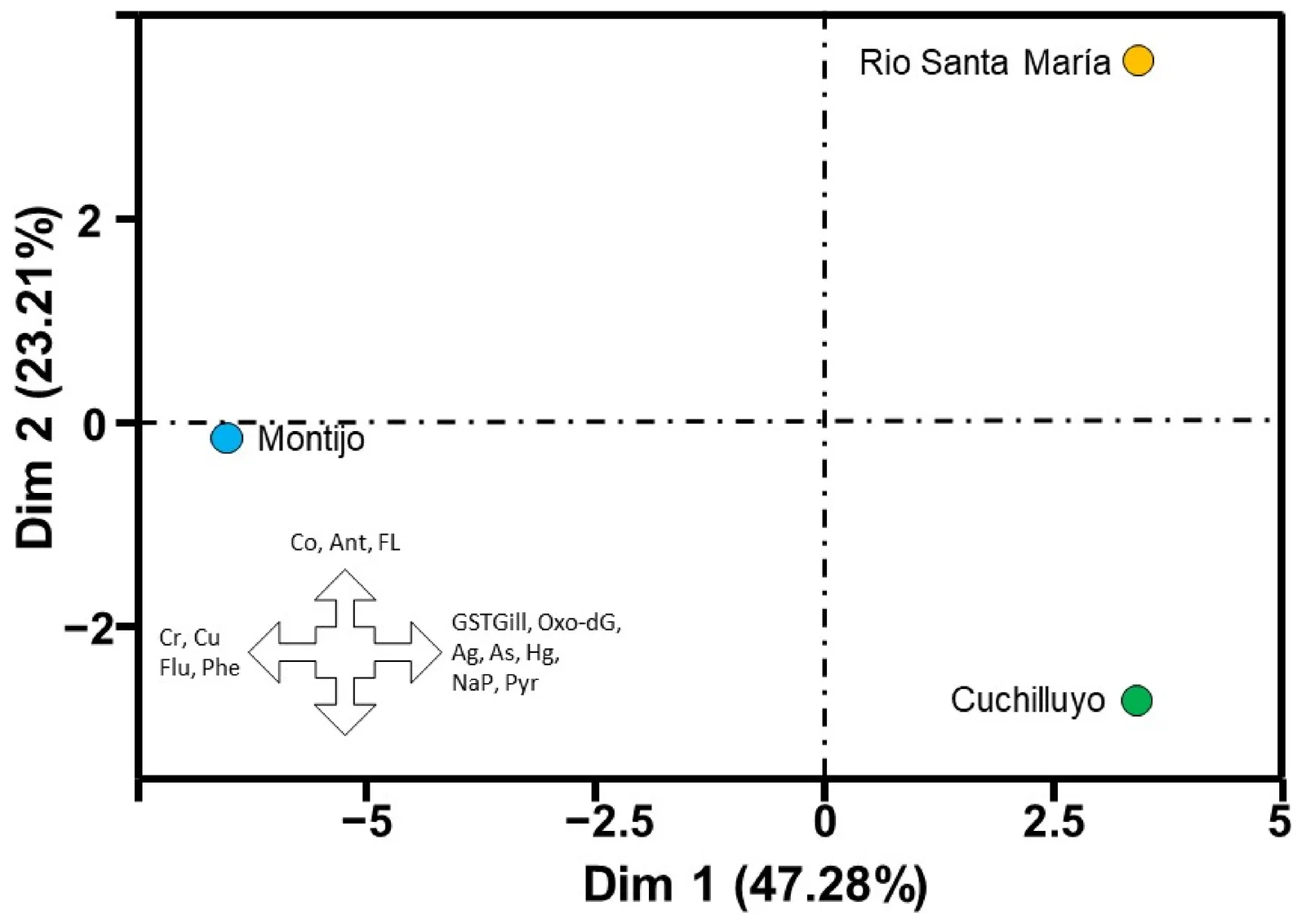
Distribution of the three sampling sites according to the Principal Component Analysis of the variables (chemical and biological) measured in the present study. The arrow symbol in the lower left corner indicates the most contributing variables to each component.

When data is integrated in a Principal Component Analysis (PCA), results indicate that the two most relevant dimensions explain 70.49% of all variances within the data (Figure 6; Table S3). More specifically, the first component explains 47.28% of all variances and clearly discriminates the two sites in the eastern area of the Azuero Peninsula (Rio Santa María and Cuchilluyo) from the more eastern area (Montijo). The presence of higher levels of potentially toxic compounds such as As, Hg, NaP, or Pyr led to higher levels of GST in the gills and higher levels of DNA oxidation measured by immunohistochemistry. On the other hand, cockles from Montijo presented higher levels of Cr, Cu, and Phe. The second dimension explains 23.21% of the variance and is linked to the presence of specific compounds such as Co, Ant, and FL, with higher levels in Rio Santa María, lower levels in Cuchilluyo, and intermediate levels in Montijo.

## 4. Discussion

Mangroves provide relevant ecosystem services, including nursery habitats for marine species, sediment stabilization, water filtration and purification, coastal protection, and carbon sequestration [10]. In the case of Panama, the Pacific coast hosts extensive mangrove forest that suffers significant anthropogenic pressures [43]. To the authors’ knowledge, there is no systematic study or program for assessing and monitoring ecosystem health status along the coast of Panama, highlighting the need and relevance of the present study. Previous studies using the cockle *Anadara tuberculosa* for the detection of pollutants in tropical coastal environments [17,20] have demonstrated its suitability as a sentinel species for the ecosystem health assessment.

Regarding chemical measurements, the metal concentrations observed herein were overall similar to or lower than those measured in other parts of the Pacific tropical coast of America. Similar studies conducted in Costa Rica [44] and Ecuador [45] with *A. tuberculosa* reported that those areas presented higher levels of Cd, Cu, Pb, or Ni and similar levels of Zn, Mn, or V, further demonstrating the species’ capacity to bioaccumulate heavy metals. On the other hand, present work values are similar to those recorded on the Pacific coast of Nicaragua for most metals [17]. The relatively high As concentrations observed in the present work, particularly in Rio Santa María and Cuchilluyo, indicate a potentially relevant As input in the area that demands further research. Indeed, these findings suggest a complex interplay between geogenic and anthropogenic factors. Specifically, the bioaccumulation in bivalves may be attributed to the weathering of natural geochemical deposits, the remobilization of As-enriched sediments, or a legacy contamination from historical arsenical pesticide applications in upstream agricultural activities (e.g., historical use of arsenical pesticides) [46]. Consequently, identifying the precise source of As is crucial for developing effective mitigation strategies and assessing long-term ecological risks in the region.

Since these cockles are widely used for human consumption, it is noteworthy that concentrations of Cd and As are within the established European thresholds [47]. However, heavy metals such as As, Cd, and Cr present cancer risks in all sites, and therefore more targeted toxicological research in the area would be required to evaluate the potential risks for both biota and human consumers. In the present study, bivalve consumption has been based on data from Ecuador [31] since currently no data is available from Panama. It should be noted that probably *A. tuberculosa* intake is higher in Ecuador due to its more developed industry in this species [48], whereas harvesting is more artisanal in Panama. Consequently, the estimated carcinogenic risk for As, Cd, and Cr will probably represent a “worst-case-scenario” condition and likely be limited to coastal populations. But the observed carcinogenic risk should not be underestimated, and detailed information seems mandatory. Reliable data on shellfish consumption in Panama would help to reduce uncertainty in the risk estimates and highlight one of the limitations of the present study.

Previous works demonstrated that heavy metal accumulation in *A. tuberculosa* may vary throughout the year, with higher values observed during the dry season [17,44]. In the present work, cockles were sampled at the beginning of May, corresponding to the end of the dry season, which may also explain the relatively high levels of As and Cd in this period. Nevertheless, the overall heavy metal content of cockles on the Panama Pacific coast remains relatively low. The Montijo site presents overall lower levels of metals of interest such as Cd, Hg, V, and Zn, indicating a less impacted area regarding metal pollution.

Although still limited, previous studies on organic pollutants present in *A. tuberculosa* from the Pacific coast indicated similar levels of PAHs compared with those observed in this study. For example, PAHs levels recorded in bivalves from Colombian mangroves ranged between 23.8 and 169.3 ng/g dry weight [20], and in the case of Nicaragua, 70–90 ng/g dry weight in the rainy season and lower in the dry season [17]. In all samples, the concentrations were below the regulatory limit for human consumption set for the European Union [47], suggesting that the sampling sites do not have substantial organic pollution. Regarding the origin of PAHs, a ratio of Flu/(Flu + Pyr) below 0.4 suggests petrogenic sources, while values higher than 0.5 suggest combustion processes [49]. In the present study, values were close to or below this 0.4 threshold, indicating a stronger influence of petrogenic sources. However, in the case of Montijo, a mixed origin cannot be discarded. However, although both are in low concentrations, the Anthracene/(Anthracene + Phenanthrene) relationship showed values above 0.1, which has been described as indicative of combustion origin, and the values close to or above 0.35 in the BaA/(BaA + Chr) ratio also indicate combustion origin on the studied sites [50,51]. According to the data, therefore, the origin of PAHs could be more petrogenic, but mixed origins could not be discarded.

Overall, the enzyme activity responses observed in *A. tuberculosa* were consistent with the chemical data. Acetylcholinesterase is a key enzyme in neuromuscular junctions, especially in muscles and nerves, and is widely used in bivalves for the assessment of neurotoxicity derived from environmental pollutants [52,53], particularly organophosphate pesticides. In general, a decrease in acetylcholinesterase activity is observed in stress conditions [53,54]. In the present study, however, values were very similar across sites, indicating that contaminants such as As, Cd, BaA, or Pyr did not exert a significant inhibition of the enzyme activity in cockles, probably indicating a limited presence of potent inhibitors such as organophosphate pesticides in the studied areas. It should be noted, however, that AChE activity may be affected by environmental factors, as previously observed in mussels where decreased enzyme activity was recorded in response to increasing salinity [55]. In the present study, it is conceivable that environmental conditions at sampling time (end of dry season with less freshwater input) may have affected the recorded enzymatic activities and that different (higher) values could be expected in the rainy season. Future monitoring in the rainy season may provide clearer insights in biochemical biomarker responses between locations.

Catalase is a well-known antioxidant enzyme and typically increases after exposure to different pollutants [28,56]. Presently, very similar levels of CAT have been measured across sites. Although it has been described that metals such as Cd or Pb [56] and PAHs [57] may enhance enzymatic CAT activity, contaminant concentrations detected in the present study do not seem to induce CAT activity at higher levels in the eastern areas of the Azuero Peninsula. In contrast, Glutathione S-transferase (GST) activity showed site-dependent variations, with lower enzymatic activity in cockles from Montijo. GST plays a key role in phase II detoxification and protection against oxidative stress. GST is also involved in the metabolism of lipophilic organic contaminant detoxification [26]. Higher levels of organic compounds such as NaP, Pyr, or BaA may explain the GST induction observed in Rio Santa María and Cuchilluyo. Accordingly, higher GST activity observed in Río Santa María and Cuchilluyo suggests increased detoxification demands associated not only with higher levels of some organic compounds but also with the presence of Cd, As, or Hg, which are known inducers of oxidative stress responses in bivalves [15,58]. The clearer trend of GST activity observed in gills compared to the digestive gland could be related to the higher presence of GST-inducing pollutants in dissolved form that bind to particulate matter [59] or to interactions between the feeding activities and GST [60] in cockles. In both cases, the trend observed suggests the presence of environmental stressors in Rio Santa María and Cuchilluyo leading to subcellular effects in *A. tuberculosa*.

Histological analysis of gonads demonstrated that individuals used for the present study were at a similar reproductive stage, suggesting a synchronized reproductive cycle in the region. Previous studies from the Montijo area showed continuous spawning of *A. tuberculosa* throughout the year [61], with high salinity conditions playing a key role [62]. In the present study, with samples collected in May, at the end of the dry season/beginning of the wet season, animals were mostly in the mature or spawning phase. Alterations in the digestive gland (including atrophy) and histopathological analyses have been widely used in environmental monitoring programs in different parts of the world [22,39,63] as useful tools to determine the overall health status of bivalves in their environment. In the present study, the atrophy levels detected in cockles were between 2 and 3, which that are values indicative of moderate damage in the digestive gland [64]. These values are consistent with the pollution loads observed herein, where most contaminants were detected at relatively low levels, but some mild alterations could still be expected in response to levels of As and, to a lesser extent, Cd, Hg, and Flu. Similarly, other authors have indicated the suitability of the atrophy of the digestive epithelium (thinning) to determine the stress level suffered by marine bivalves in different conditions [63,64,65]. Histopathological alterations such as high prevalence of hemocytic infiltrations and parasitic burdens are often recorded upon exposure to environmental stressors such as pollutants [66]. Presently, these alterations were not detected at critical levels, in agreement with the chemical profiles. Nevertheless, cockles from Cuchilluyo seemed to be more affected than individuals from Rio Santa María and Montijo, since the aforementioned lesions were more prominent at this site.

Accordingly, the 8-oxodG presents a similar trend to the histological data. The 8-oxodG is an oxidized modification of guanine that results from the presence of ROS. This modification can lead to genome instability through G-T transversions and can cause mispairing during DNA replication, inducing mutations and contributing to tumorigenesis [67,68]. Immunohistochemical stain showed a strong positive reaction in hemocytes that is probably related to the hemoglobin pigment naturally present in the genus Anadara (not hemocyanin as in the remaining bivalve genera). This high stain intensity makes the measurements through the microscope difficult and is a challenging issue for similar approaches in this species. Therefore, the measurements were focused only on the digestive epithelium, where nuclei also presented positive reactivity, indicating the presence of oxidized guanine in the DNA. This DNA damage-related biomarker has been shown to be sensitive to PAHs exposure in mussels [40,57]. In this study, higher DNA damage was recorded for individuals from Cuchilluyo, which may be related to the higher concentrations of As and Benzo(a)anthracene, in agreement with the histopathological assessment. These results are also in concordance with the biochemical responses recorded, suggesting potential oxygen radical-related stress in Rio Santa María and Cuchilluyo.

Overall, cockles from the Pacific Coast of Panama presented low-to-moderate levels of metallic (except for As) and organic pollution, as reflected in the low prevalence of histopathological alterations and the lack of reproductive alterations. Better general health status was described for individuals from Montijo, in comparison with cockles from Rio Santa María and Cuchilluyo, which presented higher levels of GST and 8-oxodG alterations. The present study demonstrates the suitability of *A. tuberculosa* as a sentinel species and initiates the application of the battery of selected biomarkers for the assessment of the general ecosystem health status on the Panama Pacific coast. In any case, further research is needed to establish baseline values for selected biomarkers, assess seasonal variability, and refine toxicological risks for the population (specific shellfish consumption data for Panama), and technical optimization (8-oxodG immunohistochemistry) is also required.

## Figures and Tables

**Table 1:** Means ± standard deviations of biometric parameters of *Anadara tuberculosa* collected in the three sampling sites.

	Rio Santa María	Cuchilluyo	Montijo
Length (cm)	4.62 ± 0.36	5.13 ± 0.49	5.78 ± 0.52
Width (cm)	3.26 ± 0.26	3.64 ± 0.35	4.22 ± 0.48
L/W ratio	1.41 ± 0.07	1.41 ± 0.09	1.37 ± 0.1

**Table 2:** Concentrations of metals (µg/g) and PAHs (ng/g) (mean ± standard deviation) in dry tissue of *Anadara tuberculosa* collected in different sites on the Pacific Coast of Panama. B.d.l. indicate values below the detection limit.

Site	Rio Santa María	Cuchilluyo	Montijo
Ag	0.01 ± 0.003	0.01 ± 0.004	0.01 ± 0.003
Al	299.67 ± 198	329.46 ± 162	33.29 ± 18.239
As	7.73 ± 1.363	9.1 ± 0.631	4.99 ± 0.642
Cd	4.27 ± 0.575	2.79 ± 1.14	0.55 ± 0.282
Co	1.16 ± 0.363	0.79 ± 0.292	0.83 ± 0.291
Cr	0.87 ± 0.052	0.81 ± 0.493	1.85 ± 0.932
Cu	3.79 ± 0.073	3.82 ± 1.054	5.83 ± 5.181
Fe	692 ± 132	715 ± 387	565 ± 196
Hg	0.11 ± 0.012	0.1 ± 0.017	0.04 ± 0.012
Mn	40.57 ± 1.219	57.12 ± 2.123	4.413 ± 1.342
Ni	0.41 ± 0.09	0.21 ± 0.05	0.46 ± 0.236
Pb	0.11 ± 0.035	0.12 ± 0.007	0.07 ± 0.072
Se	4.88 ± 1.035	2.99 ± 0.131	2.02 ± 0.64
V	1.31 ± 0.041	1.13 ± 0.579	0.65 ± 0.338
Zn	63.43 ± 2.592	58.44 ± 12.661	36.33 ± 3.864
Naphtalene	22.85 ± 5.444	21.4 ± 5.656	17.13 ± 10.293
Acenaphtene	B.d.l.	2.2 ± 0.00	B.d.l.
Fluorene	17.26 ± 16.402	16.35 ± 9.546	24.76 ± 2.944
Phenanthrene	2.65 ± 0.848	3.55 ± 1.623	6.51 ± 4.108
Anthracene	2.43 ± 3.228	0.01 ± 0.005	0.50 ± 0.005
Fluorantene	1.85 ± 0.495	1.75 ± 0.071	1.83 ± 0.814
Pyrene	4.25 ± 1.483	3.9 ± 0.707	2.73 ± 1.267
Benzo(a)anthracene	2.3 ± 0.00	6.92 ± 2.68	0.57 ± 0.208
Chrysene	1.24 ± 0.141	0.32 ± 0.282	1.33 ± 0.472
Benzo(b)fluoranthene	B.d.l.	0.53 ± 0.002	B.d.l.
Indene	0.65 ± 0.071	0.75 ± 0.003	0.36 ± 0.282
∑ PAHs	52.9 ± 24.892	57.35 ± 4.117	55.15 ± 19.238
LMW PAHs	44.56 ± 23.232	44.28 ± 6.891	49.47 ± 17.284
HMW PAHs	8.55 ± 1.732	11.82 ± 2.283	5.04 ± 2.284
LMW/HMW	5.08 ± 1.711	3.87 ± 1.342	12.22 ± 8.937
Ant/(Ant + Phe)	0.22 ± 0.098	0.13 ± 0.018	0.11 ± 0.095
Flu/(Flu + Pyr)	0.30 ± 0.012	0.32 ± 0.049	0.40 ± 0.077
BaA/(BaA + Chr)	0.66 ± 0.027	0.97 ± 0.024	0.30 ± 0.045

**Table 3:** Estimated daily intake (*EDI*), target hazard quotients (*THQ*), total hazard index (*HI*), and the target cancer risk (*TR*) of heavy metals from consuming *Anadara tuberculosa* collected in the three coastal areas of Panama.

Site		Rio Santa María	Cuchilluyo	Montijo
*EDI* (mg/kg/day)	Ag	0.0045	0.0045	0.0045
	Al	134.54	147.48	14.83
	As	0.103	0.122	0.067
	Cd	1.91	1.25	0.24
	Co	0.52	0.35	0.37
	Cr	0.38	0.36	0.83
	Cu	1.7	1.71	2.61
	Fe	310.54	320.79	253.67
	Hg	0.049	0.042	0.018
	Mn	18.21	25.63	1.98
	Ni	0.18	0.09	0.21
	Pb	0.044	0.052	0.0673
	Se	2.191	1.3402	0.907
	V	0.59	0.51	0.29
	Zn	28.45	26.22	16.29
*THQ*	As	1.109	1.305	0.717
	Cd	0.612	0.400	0.079
	Cr	0.124	0.116	0.265
	Cu	0.544	0.548	0.836
	Hg	0.016	0.014	0.006
	Pb	0.014	0.017	0.022
	Zn	9.094	8.381	5.209
*HI*		11.513	10.780	7.133
*TR*	As	0.0011	0.0013	0.0007
	Cd	0.0845	0.0551	0.0109
	Cr	0.0014	0.0013	0.0029
	Pb	2.67 × 10^−6^	3.07 × 10^−6^	4.01 × 10^−6^

**Table 4:** Estimated daily intake (*EDI*), target hazard quotients (*THQ*), total hazard index (*HI*), and the target cancer risk (*TR*) of PAHs from consuming *Anadara tuberculosa* collected in the three coastal areas of Panama.

Site		Rio Santa María	Cuchilluyo	Montijo
*EDI* (mg/kg/day)	Naphtalene	0.0102	0.0096	0.007696
	Acenaphtene	0	0.000968	0
	Fluorene	0.0077	0.00734	0.011127
	Phenanthrene	0.0011	0.00159	0.002921
	Anthracene	0.0011	4.4897 × 10^−6^	0.00023
	Fluorantene	0.0008	0.000795	0.00082
	Pyrene	0.0019	0.001746	0.001231
	Benzo(a)anthracene	0.00104	0.003102	0.00025
	Chrysene	0.000549	0.000134	0.000589
	Benzo(b)fluoranthene	0	0.000112	0
	Indene	0.000291	0.000314	8.979 × 10^−5^
*THQ*	Naphtalene	0.00049	0.00046	0.00037
	Acenaphtene	0	2.85 × 10^−10^	0
	Fluorene	0.00037	0.00035	0.00053
	Phenanthrene	0.00006	0.00008	0.00014
	Anthracene	0.00005	0.00000	0.00001
	Fluorantene	0.00004	0.00004	0.00004
	Pyrene	0.00009	0.00008	0.00006
*HI*		0.0011	0.00101	0.00115
*TR*	Chrysene	3.84 × 10^−9^	9.43 × 10^−10^	4.129 × 10^−9^

## Data Availability

The original contributions presented in this study are included in the article/Supplementary Materials. Further inquiries can be directed to the corresponding author.

## Supplementary Materials

The following supporting information can be downloaded at: https://www.mdpi.com/article/10.3390/toxics14080710/s1, Table S1: Overall model of the Bayesian ANOVA indicating mean values, Bayesian factor10 and evidence based on [41]. Table S2: Pairwise comparison of the variables with higher BF and evidence based on [41]. Table S3: Eigenvalues and variance percentage explained by the main components after PCA analysis.

## Abbreviations

The following abbreviations are used in this manuscript:

8-oxodG	8-Oxo-7,8-dihydro-2′-deoxyguanosine
AChE	Acetylcholinesterase
Ant	Anthracene
AS	Area Surface
AT	Mean Exposure Duration for non-carcinogens
BaA	Benzo(a)anthracene
BaP	Benzo(a)pyrene
BbFL	Benzo(b)fluoranthene
BkFL	Benzo(k)fluoranthene
BW	Average Body Weight
CAT	Catalase
Chr	Chrysene
*CSF*	Carcinogenic Slope Factor
DBA	Dibenzoanthracene
DTNB	5,5′-ditiobis-(2-nitrobenzoic) acid
ED	Exposure Duration
*EDI*	Estimated Daily Intake
EF	Exposure Frequency
EHU	University of the Basque Country
EPA	Environmental Protection Agency
FIR	Daily Intake of Bivalves
Flu	Fluorantene
G	Guanine
GST	Glutathione S-transferase
*HI*	Hazard Index
HMW	Heavy Molecular Weight
IHC	Immunohistochemistry
Ind	Indene
LMW	Light Molecular Weight
MAG	Average Grey Value
OD	Optical Density
PAH	Polycyclic Aromatic Hydrocarbon
PBS	Phosphate-Buffered Saline
PCA	Principal Componen Analysis
Phe	Phenanthrene
PMS	Post-Mitochondrial Supernatant
Pyr	Pyrene
RfD	Oral Reference Dose
T	Thymine
*THQ*	Target Hazard Quotient
TR	Cancer Risk
