# Supplementary material for: Health Status Assessment of Panamanian Pacific Coastal Areas Using Multi-Level Biomarkers in the Mangrove Cockle Anadara tuberculosa
Source: Toxics. 2026 Aug 12;14(8):710. doi: 10.3390/toxics14080710 (PMC13517178; doi:10.3390/toxics14080710)
Supplement: Supplementary file 1 [file toxics-14-00710-s001.zip › toxics-4322701-supplementary.pdf]

Supplementary Table S1. Overall model of the Bayesian ANOVA indicating mean values, Bayesian factor10 and evidence based on [41].

Variable	Mean Rio Santa María	Mean Cuchilluyo	Mean Montijo	BF10	Evidence
**Length**	4.62	5.13	5.78	4.0E06	Very strong
**Width**	3.26	3.64	4.22 ± 0.48	8.0E06	Very strong
**L/W ratio**	1.41	1.41	1.37	0.065	Strong H0
**Ag**	0.01	0.01	0.01	0.47	Anecdotal
Al	299.67	329.00	33.29	1.38	Anecdotal
**As**	7.73	9.10	4.99	4.57	Moderate
**Cd**	4.27	2.79	0.55	16.95	Strong
**Co**	1.16	0.79	0.83	0.69	Anecdotal
**Cr**	0.87	0.81	1.85	1.06	Anecdotal
**Cu**	3.79	3.82	5.83	0.49	Anecdotal
**Fe**	692	715	565	0.49	Anecdotal
**Hg**	0.11	0.1	0.04	121.18	Very strong
**Mn**	40.57	57.12	4.41	10080.66	Very strong
**Ni**	0.41	0.21	0.46	0.79	Anecdotal
**Pb**	0.11	0.12	0.07	0.55	Anecdotal
**Se**	4.88	2.99	2.02	5.28	Moderate
**V**	1.31	1.13	0.65	0.93	Anecdotal
**Zn**	63.43	58.44	36.33	7.46	Moderate
**Naphtalene**	22.85	21.4	17.13	0.52	Anecdotal
**Fluorene**	17.26	16.35	24.76	0.58	Anecdotal
**Phenanthrene**	2.65	3.55	6.51	0.72	Anecdotal
**Anthracene**	2.43	0.01	0.50	0.65	Anecdotal
**Fluorantene**	1.85	1.75	1.83	0.43	Anecdotal
**Pyrene**	4.25	3.9	2.73	0.71	Anecdotal
**Benzo(a)anthracene**	2.3	6.92	0.57	4.04	Moderate
**Chrysene**	1.24	0.32	1.33	1.64	Anecdotal
**Indene**	0.65	0.75	0.36	1.17	Anecdotal
**AChE Gill**	9.14	5.53	10.7	0.39	Anecdotal
**CAT Gill**	0.082	0.080	0.084	0.054	Strong H0
**GST Gill**	62.9	68.1	29.3	21.74	Strong
**GST DG**	182.9	123.3	51.0	21.83	Strong
**Gonad index**	4.00	3.53	3.86	0.089	Strong H0
**Atrophy**	2.67	2.56	2.43	0.074	Strong H0
**8-OxodG**	0.052	0.063	0.042	12.65	Strong

Supplementary Table S2. Pairwise comparison of the variables with higher BF and evidence based on [41].

Variable	Comparison	ΔM	95% CrI	BF10	Evidence
**Length**	Rio Santa María-Cuchilluyo	-0.52	[-0.80, -0.23]	31.95	Strong
**Length**	Rio Santa María-Montijo	-1.16	[-1.46, -0.86]	4.0E6	Extreme
**Length**	Cuchilluyo-Montijo	-0.64	[-0.96, -0.33]	185.2	Strong
**Width**	Rio Santa María-Cuchilluyo	-0.37	[-0.58, -0.17]	36.6	Strong
**Width**	Rio Santa María-Montijo	-0.96	[-1.21, -0.71]	1.13E6	Extreme
**Width**	Cuchilluyo-Montijo	-0.58	[-0.85, -0.32]	535.6	Strong
**As**	Cuchilluyo–Montijo	4.102	[3.250, 4.953]	6.660	Moderate
**Cd**	Rio Santa María–Montijo	3.717	[3.233, 4.353]	37.210	Strong
**Hg**	Rio Santa María–Montijo	0.070	[0.060, 0.080]	57.628	Strong
**Hg**	Cuchilluyo–Montijo	0.055	[0.050, 0.060]	14.227	Strong
**Mn**	Rio Santa María–Cuchilluyo	-16.533	[-18.800, -14.267]	11.981	Strong
**Mn**	Rio Santa María–Montijo	36.153	[34.590, 37.717]	1222.970	Strong
**Mn**	Cuchilluyo–Montijo	52.687	[50.390, 54.983]	102.929	Strong
**Se**	Rio Santa María–Montijo	2.860	[1.720, 3.877]	4.292	Moderate
**Zn**	Rio Santa María–Montijo	27.067	[23.000, 31.100]	34.796	Strong
**Benzo(a)anthracene**	Cuchilluyo–Montijo	1.733	[1.500, 1.900]	23.381	Strong
**GST Gill**	Cuchilluyo–Montijo	38.78	[9.33,68.48]	7.19	Moderate
**GST DG**	Rio Santa María–Montijo	131.8	[17.6,245]	4.45	Moderate
**8-OxodG**	Cuchilluyo–Montijo	0.0216	[0.0072,0.0358]	11.46	Strong

Supplementary Table S3. Eigenvalues and variance percentage explained by the main components after PCA analysis.

Component	Eigenvalue	% of Variance	Cumulative %
1	10.87422	47.279%	47.279%
2	5.33719	23.205%	70.484%
3	3.22167	14.007%	84.492%
4	1.94639	8.463%	92.954%
5	1.62054	7.046%	100.000%
